# Folate Receptor Alpha Autoantibodies in the Pediatric Acute-Onset Neuropsychiatric Syndrome (PANS) and Pediatric Autoimmune Neuropsychiatric Disorders Associated with Streptococcal Infections (PANDAS) Population

**DOI:** 10.3390/jpm14020166

**Published:** 2024-01-31

**Authors:** Lindsey Wells, Nancy O’Hara, Richard E. Frye, Neeshi Hullavard, Erica Smith

**Affiliations:** 1 Lindsey Wells ND LLC, Wilton, CT 06897, USA; lwells@lindseywellnd.com; 2 Nancy O’Hara and Associates LLC, Wilton, CT 06897, USA; nhohara@drohara.com; 3 Autism Discovery and Treatment Foundation, Phoenix, AZ 85050, USA; 4 Massachusetts General Hospital, Boston, MA 02114, USA; neeshi.hullavarad@gmail.com; 5 Natural Pediatrics of CT, Stamford, CT 06905, USA; drsmith@ericasmithnd.com

**Keywords:** PANS, folate receptor alpha antibodies, PANDAS, OCD, autism spectrum disorder, tics, leucovorin

## Abstract

The folate receptor alpha autoantibodies (FRAAs) are associated with cerebral folate deficiency (CFD) and autism spectrum disorder (ASD). Both of these syndromes have overlapping characteristics with Pediatric Autoimmune Neuropsychiatric Disorders Associated with Streptococcal Infections (PANDAS) and Pediatric Acute-Onset Neuropsychiatric Syndrome (PANS). Thus, we propose that the FRAAs may contribute to the symptomatology of PANS/PANDAS. To test this hypothesis, 1 mL of serum from 47 patients (age range = 6–18 years old) clinically diagnosed with PANS/PANDAS was sent to Vascular Strategies (Plymouth Meeting, PA, USA) for analysis of FRAAs. Moreover, 63.8% of PANS/PANDAS patients (male = 15; female = 15) were found to have either the blocking and/or blinding FRAAs, with 25 (83.3%; male = 14; female = 11) having binding FRAAs, two (6.7%; all female = 2) having blocking FRAAs, and 3 (10%; male = 1; female = 2) having both binding and blocking. Furthermore, surprisingly, ASD was associated with a 0.76 lower binding titer (*p* = 0.02), and severe tics were associated with a 0.90 higher binding titer (*p* = 0.01). A case of a FRAA-positive patient is provided to illustrate that a treatment plan including leucovorin can result in symptom improvement in patients with PANS/PANDAS who are FRAA-positive. These data, for the first time, demonstrate that PANS/PANDAS is associated with FRAAs and suggest folate metabolism abnormalities may contribute to PANS/PANDAS symptomatology. Further studies investigating the therapeutic nature of leucovorin in the treatment of PANS/PANDAS are needed. Such studies may open up an alternative, safe, and well-tolerated treatment for those with the PANS/PANDAS diagnosis.

## 1. Introduction

Neuropsychiatric Disorders Associated with Streptococcal Infections (PANDAS) is an autoimmune disorder impacting neurological function in prepubescent children [1]. It presents with an acute and dramatic onset of obsessive-compulsive disorder (OCD) and/or tic disorder, neurologic abnormalities with prepubescent onset, and an episodic waxing and waning course associated with Group A streptococcal (GAS) infection [2]. In PANDAS, the GAS infection induces encephalitis that interferes with brain function in areas such as the basal ganglia [3]. Pediatric Acute-Onset Neuropsychiatric Syndrome (PANS) is a similar disorder defined as a sudden dramatic overnight onset of OCD and/or severe restrictive eating with at least two comorbid symptoms such as anxiety, emotional lability, developmental regression, sensory and motor abnormalities, urinary symptoms, sleep disturbance, tics, and/or a decline in academic performance. Although the criteria do not specify a specific etiological trigger, it is speculated in many cases that infectious agents such as *Mycoplasma pneumoniae* and influenza, as well as other viruses, act as triggers [4]. It has been speculated that infectious agents with prolonged colonization periods are likely to cause PANS [4]. PANS encapsulates disorders that cannot be explained by a known neurological or medical disorder [1]. 

Determination of PANS/PANDAS requires a clinical diagnosis based on thorough consideration of family, medical, and psychiatric history, physical and psychiatric exams, and infectious and autoimmune disease evaluations [1,4]. Laboratory work can support diagnosis; however, as the name suggests, diagnosis of PANS/PANDAS is determined by the acute onset of symptoms. Therefore, in the face of a clear acute onset of symptoms and negative laboratory findings that rule out other potential diagnoses, PANS or PANDAS is entertained. PANS/PANDAS treatment follows a three-pronged approach. First, antimicrobials are used to treat any underlying infections. Second, cognitive-behavioral therapy and pharmacological interventions are used for psychiatric and behavioral support. Lastly, anti-inflammatory agents, corticosteroids, intravenous immunoglobulin, and nutrient supplementation are used for immunomodulatory support [5].

Not all patients respond adequately to this three-pronged approach to the treatment of PANS/PANDAS, suggesting that their symptoms may be either driven by another etiology or that there is a comorbid condition that also requires treatment. One disorder that is being increasingly recognized is cerebral folate deficiency (CFD). CFD has been associated with many symptoms that overlap PAN/PANDAS, such as acute onset, movement disorders such as tics, cognitive impairment, developmental delays, and autism spectrum disorder (ASD) [6,7]. CFD has been reported in disorders that overlap PANS/PANDAS symptomatology, such as refractory depression and suicidal ideations [8,9] and schizophrenia [10]. Many individuals with CFD have ASD, a disorder that includes OCD and repetitive behaviors similar to PANS/PANDAS symptomatology [11]. Thus, it may be possible that individuals with PAN/PANDAS may be affected by the same mechanism that causes CFD. This has never been investigated before.

Folate is a water-soluble B-vitamin necessary for cerebral metabolism and function throughout development, adolescence, and adulthood [12,13,14]. In patients with CFD, normal levels of 5-methyltetrahydrofolate (5MTHF) are found in serum despite below-normal concentrations of 5-methyltetrahydrofolate (5MTHF) in the cerebrospinal fluid (CSF) [15,16]. This is caused by dysfunction of the folate receptor alpha (FRɑ), the main transporter of folate into the brain [16]. In healthy conditions, folate binds to the FRɑ, which then undergoes endocytosis to actively transport folate across the blood–brain barrier. 

One of the main causes of FRɑ dysfunction are FRɑ autoantibodies (FRAAs), which bind to the FRɑ and inhibit folate transport into the CSF [6,17]. Two FRAAs have been described, i.e., the blocking and binding FRAAs. The blocking FRAA binds specifically to the site where folate binds to the FRα, thereby preventing folate from binding to the FRɑ, while the binding FRAA binds to other regions of the FRɑ and interferes with its ability to function optimally.

To determine the presence of CFD, standard practice utilizes lumbar punctures to measure the 5MTHF concentration in the CSF. Since the lumbar puncture is invasive [12] and generally requires a general anesthetic [14], it has become common practice to measure FRAAs using the folate receptor autoantibody test (FRAT). Previous studies have found the serum titers of FRAAs in patients with CFD and ASD correlate with the CSF concentration of 5MTHF, validating the use of FRAAs as a proxy for folate abnormalities in the brain [12,15,18]. The drawback to this approach is that CFD cannot be diagnosed with only serum FRAA titers. However, since the treatment of central folate abnormalities, leucovorin, is very safe, the FRAAs in conjunction with a treatment trial are believed to have a lower risk than performing a lumbar puncture [12]. 

Studies suggest that central folate abnormalities play an essential role in the pathogenesis and exacerbation of ASD [12,17]. Successful treatment of CFD and FRAA-positive ASD patients provides a basis for believing that central folate abnormalities are a treatable condition. Similar to ASD, it is believed that the etiology of the majority of PANS/PANDAS cases includes triggers from environmental factors, including factors that induce the production of FRAAs [17]. A meta-analysis estimates the prevalence of FRAAs in ASD to be 71% [11]. Leucovorin treatment (folinic acid) in FRAA-positive ASD patients results in improvements in ASD symptoms, including speech, communication, and repetitive behaviors [18]. 

Based on the overlap between PANS/PANDAS symptoms and CFD/ASD, such as repetitive behaviors, tics, depression, neurodevelopmental regression, and speech disfluency, we propose that FRAAs may play a role in the symptomatology of PANS/PANDAS. If FRAAs were found in patients with PANS/PANDAS, leucovorin or other reduced folate may provide symptomatic relief for many of the symptoms that often remain refractory to treatment. This is the first study to address this question. 

## 2. Materials and Methods

### 2.1. Subjects

In total, 47 children and adolescents (age range = 6 to 18; age mean = 11 years; male = 29; female = 18) that met the criteria for PANS/PANDAS were included in this study. PANDAS is defined by the prepubescent acute and dramatic onset of OCD and/or tic disorder, along with neurologic abnormalities and an episodic waxing and waning course associated with GAS infection [2]. PANS is defined as the sudden dramatic overnight onset of OCD and/or severe restrictive eating with at least two comorbid symptoms such as anxiety, emotional lability, developmental regression, sensory and motor abnormalities, urinary symptoms, sleep disturbance, tics, and/or a decline in academic performance [1].

These patients were evaluated, tested, and diagnosed by Lindsey Wells, ND, and Nancy O’Hara, MD, after a thorough workup to eliminate other cases. Associated symptoms such as OCD and ASD were diagnosed by a developmental-behavioral pediatrician, neurologist, or psychiatrist. Laboratory values and symptomatology were abstracted from records by the treating physicians into a deidentified database for analysis. This procedure was determined to be exempt under 45 CFR § 46.104(d)(4) by the WCG IRB (Puyallup, WA, USA). Symptoms of anxiety, OCD, tics, depression, and attention deficit-hyperactivity disorder (ADHD) were rated on four levels: none, mild, moderate, and severe. ASD was rated as present or not present.

### 2.2. Folate Receptor Alpha Autoantibody Assay

Approximately 2–4 mL of blood was drawn into a serum-separating tube, and the serum was separated by centrifugation. Moreover, 1 mL of serum was transferred to a transport tube and shipped. The FRAA assay was performed by Vascular Strategies (Plymouth Meeting, PA, USA) through their clinical laboratory improvement amendments-certified pathway. The assay provides three results, i.e., the binding and blocking titers in the serum and whether the soluble folate-binding proteins are present. 

Soluble folate-binding proteins are proteins in the blood that bind folate, thereby making folate less available. None of the patients manifested soluble folate-binding proteins in their serum; this is not discussed further. 

The binding titer measures the binding of IgG antibodies to the FRɑ using an enzyme-linked immunosorbent assay (ELISA) as previously described [19]. Both positive and negative controls are run with the assay, and the titer is reported in optical density units. 

The blocking assay specific for binding IgG is used to measure binding FRAAs as previously described [19]. The blocking assay uses an in vitro assay to measure the amount of autoantibody that is specifically blocking the binding site for folate in the FRɑ. In this assay, the amount of radiolabeled folate displaced from the FRɑ when the patient’s serum is added is measured and reported in pmol blocked per ml as previously described [19]. 

The blocking titer has been shown to correlate with CSF levels of 5MTHF [12], and patients with blocking and/or binding titers may have unique behavioral and biochemical characteristics [18]. The presence or absence of the blocking and binding FRAAs is predictive of the response to leucovorin, at least in patients with ASD [11]. 

### 2.3. Statistical Analysis

To determine the relationship between symptomatology and FRAA titers, first a multivariable binary logistic regression (LR) model was used for dichotomous variables such as ASD (ASD/no ASD), and a multivariable ordinal (multinomal) LR was used for symptomatic variables with multiple levels (none, mild, moderate, or severe). Symptomatic variables include ASD, anxiety, OCD, tics, depression, and ADHD symptoms. Demographic variables, including sex and age, were initially included in the analyses but were found not to be significant and were subsequently removed. In these analyses, binding and blocking FRAA titers were predictor variables. Once a relationship between the symptomatology and FRAA titers was found, a linear regression equation was developed with the blocking or binding FRAA titers as the dependent variable and the symptoms identified as related to the FRAAs as the predictor variables. This provided an analysis of the relative influences of each symptom on the FRAA titer as well as testing for interactions between the symptoms. This two-step approach was used to limit the number of variables in the models to prevent overfitting. An alpha of 5% was used, and all models were simplified to remove non-significant, non-dependent variables. The odds ratio (OR) and linear regression coefficients (β) are presented with their 95% confidence interval.

## 3. Results

### 3.1. Patient Characteristics

Table 1 provides the demographics of the patients. The majority of patients were either positive for only the binding FRAA (53%), or they were negative for both FRAAs (40%). The patients were of similar ages, although those that were positive for both the binding and blocking FRAAs were slightly younger. Those positive for FRAAs tended to be more female than those negative for FRAAs. The population was mostly white across all groups. Those that were positive for both the binding and blocking FRAAs had a higher rate of being prescribed ADHD medications and having allergies.

### 3.2. Prevalence of FRAAs

**Figure 1:**
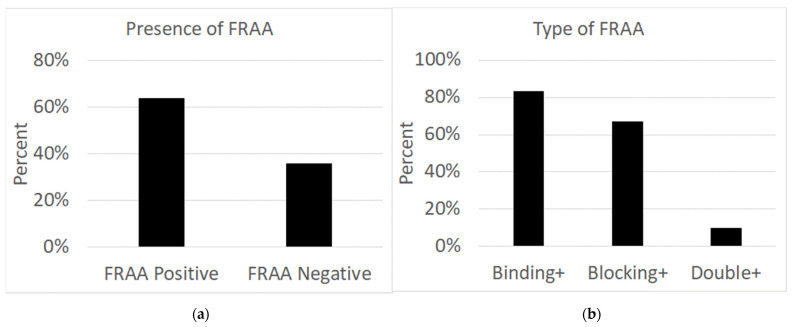
Prevalence of folate receptor alpha antibodies in patients. (**a**) Positive or negative status of all study participants. (**b**) Prevalence of specific FRAA for those that were FRAA positive. Double+ signifies positivity for both blocking and binding FRAA.

As seen in Figure 1, out of 47 patients, 30 individuals (63.8%; male = 15; female = 15) had folate receptor autoantibodies in their serum, while 17 individuals (36.2%; male = 14; female = 3) did not have any autoantibodies in their serum. Of the 30 patients with autoantibodies, 25 (83.3%; male = 14; female = 11) patients had binding autoantibodies, two (6.7%; male = 0; female = 2) patients had blocking autoantibodies, and three (10%; male = 1; female = 2) had both binding and blocking autoantibodies.

### 3.3. Association of Symptoms with FRAAs

The presence or absence of ASD was found to be significantly related to the binding FRAA, such that the presence of ASD was more likely to be associated with a lower binding FRAA [Χ^2^ (1) = 8.67, *p* < 0.01; OR = 0.13 (0.01, 0.93)]. Tics were also found to be significantly related to the binding of FRAA, such that those having tics were more likely to have a high FRAA binding titer [Χ^2^ (1) = 6.24, *p* = 0.01; OR = 3.53 (2.97, 4.20)]. For tics, this was most significant for severe tics [Χ^2^ (1) = 18.62, *p* < 0.001 OR = 13.1 (4.01, 42.52)] and borderline significant for moderate tics [Χ^2^ (1) = 3.29, *p* = 0.07; OR = 2.16 (0.93, 5.01)]. 

To examine the effect of ASD and tics simultaneously on the blocking FRAA, the tic variable was dichotomized as to whether severe tics were present or absent. ASD was found to be significantly related to binding titers [Χ^2^ (1) = 5.40, *p* = 0.02; β = −0.76 (−0.10, −1.41)], with those with ASD demonstrating a binding FRAA titer that was 0.76 OD lower than those without ASD. Tics were also significantly related to binding FRAA titers [Χ^2^ (1) = 6.30, *p* = 0.01; β = 0.90 (0.18, 1.62)], with those having severe tics having a binding FRAA titer that was 0.90 OD higher than those not having severe tics. Nine patients had ASD, with three of them having tics, but none of them having severe tics.

### 3.4. Case Study of a FRAA-Positive PANS Patient

An 18-year-old male was previously diagnosed with PANS and autoimmune encephalitis at 13 years of age due to a sudden onset of anxiety, refusing to go to school, frequent urination, OCD, and repeated strep infections. Antibiotic treatment for GAS resulted in a 30% improvement in symptoms. IVIG treatment resulted in a 60–70% improvement in symptoms. Symptoms were manageable for several years with prophylactic antibiotics. The patient then developed mycoplasma pneumonia, resulting in an exacerbation of anxiety and OCD. Plasmapheresis decreased mycoplasma titers, but the titers remained elevated. Two years later, he developed an Epstein-Barr virus (EBV) infection, which was treated with antivirals and other supplements. Since then, he has been unable to tolerate interventions such as antibiotics, herbals, and supplements due to negative reactions and continued to experience severe anxiety and OCD. Other interventions included cognitive behavioral therapy, antipsychotics, and selective serotonin reuptake inhibitor medications. Previous medical and surgical history also included a tonsillectomy and adenoidectomy at 2 years of age, frequent GAS infections, influenza, and a ruptured appendix at 11 years of age. Due to sensitivities, he eliminated dairy and gluten from his diet. The patient had trouble leaving the house, taking care of personal hygiene, and began having insomnia and obsessive thoughts that mainly occurred in his dreams.

Upon presentation to our clinic, medications included propranolol (10 mg/day) and fluvoxamine (50 mg/day). The diagnosis of PANS was made due to the acute onset of symptoms at 13 years of age, including anxiety, OCD, sleep disturbances, recurrent GAS infections, improvement with antibiotics/plasmapheresis/IVIG in the past, lab evidence of mycoplasma and viruses, and low immunoglobulins. Continued therapy was recommended, and he began working with a cognitive-behavioral therapist three times a week. Further recommendations were made based on medical history and laboratory work.

Important findings from laboratory work included persistent mycoplasma IgG, IgM, and MTHFR C677T homozygous defects, as well as low normal levels of vitamin D (32) and vitamin A (30), IgG subclass 2 (118), red blood cell (RBC), magnesium (3.8), and RBC zinc (7.67). Interventions tried without improvements in symptoms included antivirals, immune, mitochondrial, and metabolic support. Vitamins D and A, zinc, and magnesium were added to treat mild deficiencies. The patient also had a positive binding FRAA autoantibody. To address this, leucovorin (5 mg/day) was added. The patient was instructed to increase leucovorin weekly by 1 pill (5 mg) up to 30 mg per day (6 pills per day) as tolerated and helpful. Additionally, further antimicrobials were prescribed to treat mycoplasma. However, the patient did not take them due to a past history of symptom exacerbations.

Upon implementation of the above, OCD and anxiety improved by 50%, and daily function improved by 70%. Dreams were no longer obsessive, his ability to take care of hygiene improved, and his personality started to come back, but social anxiety persisted. After further implementation of leucovorin and despite persistent mycoplasma titers and no antimicrobial therapy, the patient’s symptoms improved by 90%. He noted that when he neglected to take his leucovorin, his anxiety and OCD symptoms worsened. With leucovorin treatment, the patient is currently excelling academically at a four-year university with honors, has friends, enjoys multiple hobbies, lives independently, and no longer experiences significant social anxiety or OCD.

## 4. Discussion

FRAT was conducted on 47 children and adolescents clinically diagnosed with PANS/PANDAS to determine the presence of serum FRAAs. An FRAA prevalence of 63.8% was found, similar to the prevalence found in ASD [11]. Patients with CFD and/or ASD positive for FRAAs respond well to leucovorin, a folate that can circumvent the block of the FR*ᾳ*. Thus, similarly, it is possible that FRAA-positive PANS/PANDAS patients may benefit from the same treatment. We provided a case study of one patient that showed significant improvement in OCD and anxiety with leucovorin, but we have found that other PANS/PANDAS patients also do very well when treated with leucovorin or 5-methyltetrahydrofolate (5-MTHF). 

Blocking FRAA titers has been shown to correlate with CSF folate concentrations in CFD [17] and ASD [13]. In a study, FRAAs were found in 89% of children with CFD but were not found in any children without neurological or developmental disorders [20]. However, FRAAs have been detected in unaffected siblings and parents of children with ASD [21]. In contrast, FRAAs have only been found in 15% of normal children without ASD siblings [11]. Therefore, we can assume that the presence of FRAAs in non-PANS/PANDAS patients is possible and emphasize that the FRAAs are not diagnostic of any specific disorder but instead serve as an adjunctive measure to direct a treatment approach.

The FRAT differentiates between binding and blocking autoantibodies. In this study, 83.3% of PANS/PANDAS patients that tested positive for FRAAs in their serum had binding autoantibodies, and 6.7% had blocking autoantibodies. While there is no research on the effect of autoantibody type in PANS/PANDAS, binding and blocking FRAAs have been associated with different physiologies and phenotypes in children with ASD [18]. It was found that children with ASD that were positive for the binding FRAA had significantly higher B12 concentrations in their serum compared to children with ASD that were negative for the binding FRAA [18]. High B12 levels in the serum may indicate inadequate cellular uptake of B12, which may exacerbate ASD symptoms. This assumption supports the finding that ASD patients with the binding FRAA had poorer social skills than those with the blocking FRAA [18]. Children with ASD who were positive for the blocking FRAA had better physiological and behavioral profiles compared to children with ASD who were positive for the binding FRAA and or who were FRAA negative. The blocking FRAA was also associated with better redox metabolism and inflammation markers as compared to those negative for the blocking FRAA [18]. More research is necessary to see if the type of FRAAs, binding or blocking, impacts PANS/PANDAS cellular mechanisms and symptoms.

When bioavailable folate concentrations are low, folate enters the CSF by binding to the FR*ᾳ* on the epithelium of the choroid plexus and undergoes endocytosis. FR*ᾳ* has a high affinity for folic acid and 5-MTHF [12,22]. However, when folate concentrations are high, folate can also cross the blood–brain barrier using the reduced folate carrier (RFC) [22], which is a transmembrane protein found on the basolateral and apical sides of the epithelium of the choroid plexus [23]. The RFC only transports reduced folates such as 5-MTHF and leucovorin but does not transport oxidized folates such as folic acid [19]. The RFC provides another mechanism for folate to enter the CSF. In CFD, when the FR*ᾳ* is dysfunctional, high doses of leucovorin are needed because of the low affinity of the RFC for folate [18]. This is also reflected in our case study, in which leucovorin doses were increased according to patient tolerance and resulted in improved symptoms. While further research on the effects of leucovorin in PANS/PANDAS patients is necessary, it should still be considered as mood support for those patients due to evidence that it helps with depression, cognitive function, anxiety, and OCD.

Methylenetetrahydrofolate reductase (MTHFR) is necessary for folate metabolism [24]. Polymorphisms of the MTHFR gene result in a decrease in MTHFR enzyme activity and have been associated with psychiatric disorders such as ADHD, ASD, depression, schizophrenia, and bipolar disorder. The clinical presentation of MTHFR deficiency includes gait disorder, cognitive decline, epileptic syndromes, encephalopathy, and psychotic symptoms [25]. Metabolic treatment has been shown to stabilize patients or improve their symptoms. In a study with patients clinically diagnosed with depression and no improvement after using three different antidepressants at maximum dosage for adequate duration, it was found that 36% of patients had CFD [26]. When their antidepressant medication was supplemented with leucovorin for at least 6 weeks, symptoms improved, ranging from marginal to substantial improvement.

In the case study, the patient showed improvement in OCD symptoms with leucovorin supplementation and noted worsening of symptoms when the dose was missed. There is conflicting evidence suggesting that folate deficiency underlies OCD [27,28,29]. When looking at individuals with OCD compared to neurotypical controls, OCD individuals had lower serum folate levels and higher homocysteine levels [27]. However, B12 deficiency is known to exacerbate OCD symptoms [28,29]. As previously stated, the binding FRAA in children with ASD (the case study was positive for binding autoantibodies) was associated with higher serum B12 levels; therefore, the OCD-like symptoms in PANS/PANDAS may be associated with B12 deficiency as well, considering that B12 and folate are involved in the same metabolic pathway. Because ASD and PANS/PANDAS have different overlapping but distinct symptomatology, it could be that CFD manifests differently in the two disorders. Our case also showed improved anxiety. Folate deficiency has been seen to cause an increase in anxiety in a rat model. Rats that were given FRAAs during gestation and pre-weaning displayed anxiety behaviors as adults, suggesting that folate is implicated in anxiety-like behaviors as well [30]. Taken together, these findings show that supplementing with a reduced folate, like leucovorin or 5-MTHF, in children diagnosed with PANS/PANDAS may improve symptoms.

This study has several limitations, including the limited sample size, the retrospective nature of the data collection, the use of a sample for convenience, and the lack of blinding by the assessor. However, the physicians who diagnosed the children were not aware of their FRAA status before the test was performed, providing some blinding to the outcome variables. Clearly, a prospective study with blinded assessments is needed to confirm this compelling data. 

## 5. Conclusions

The goal of this paper is to propose FRAT as an adjunctive tool to support PANS/PANDAS treatment. Our findings revealed a FRAA prevalence of 63.8% in our study population. We also included a case study of an 18-year-old male with PANS who had debilitating OCD and anxiety and had significant improvements in symptoms with the introduction of leucovorin to address a positive binding FRAA. This case study was of particular interest because this young man was not able to tolerate antimicrobial treatment, which is critical in the treatment of PANS. However, once his central folate metabolism abnormalities were addressed, he was better able to tolerate antimicrobials to treat underlying infections contributing to PANS. This is the first report to our knowledge connecting FRAAs with PANS/PANDAS. Our results suggest that children and adolescents with PANS/PANDAS should be screened with FRAT, and if positive FRAAs are found, appropriate treatment should be considered for symptomatic support. However, more research regarding this topic needs to be carried out in the PANS/PANDAS population. Systematic clinical studies demonstrating the effectiveness of leucovorin in FRAA-positive PANS/PANDAS patients would be very helpful to support treatment recommendations with leucovorin. 

## Figures and Tables

**Table 1:** Participant characteristics.

	Blocking+ and Binding+	Binding+	Blocking+	Negative	Overall
Number of patients (%) Age (SD) in years	3 (6%) 8.6 (1.5)	25 (53%) 11.28 (3.5)	2 (4%) 12.5 (5.3)	17 (40%) 11.29 (4.3)	47 11.1 (3.7)
Sex (% Male)	33%	56%	0%	82%	61%
White/Black/Asian	100%/0%/0%	96%/14%/0%	100%/0%/0%	100%/0%/0%	98%/2%/0%
Binding (O.D.) Mean (SD)	1.36 (0.94)	1.45 (0.91)	0.0	0.0	0.90 (1.00)
Blocking (pmol/mL) Mean (SD)	2.12 (1.11)	0.0	1.78 (0.26)	0.0	0.23 (0.68)
Anti-fungal	0%	4%	0%	0%	2%
Antiviral	0%	4%	0%	5%	4%
Antibiotics	0%	44%	100%	47%	44%
Stimulants/Alpha Adenergics	33%/33%	0%/8%	0%/0%	5%/11%	4%/8%
Allergy and Asthma/GI Meds	33%/0%	4%/0%	0%/0%	29%/5%	14%/2%
Anti-Inflammatory and Immunomodulatory	33%/0%	24%/12%	0%/0%	5%/5%	17%/8%
Antipsychotics/Other Psychotropics	0%/0%	4%/0%	50%/0%	11%/0%	8%/0%
SSRIs /Beta-Blockers	0%/0%	16%/4%	50%/0%	29%/0%	23%/2%
Antiepileptic Meds	4%	0%	0%	0%	2%
Thyroid Meds/Other Hormones	0%/0%	0%/0%	0%/0%	0%/0%	0%/0%

## Data Availability

Deidentified data is available upon request.
